# Energy-resolved fast-neutron radiography using an event-mode neutron imaging detector

**DOI:** 10.1038/s41598-024-81412-z

**Published:** 2024-12-16

**Authors:** Alexander Wolfertz, Adrian Losko, Alexander M. Long, Sophia Brodish, Aaron E. Craft, Anton Khaplanov, Sven C. Vogel, Ronald O. Nelson, Stephen A. Wender, Anton Tremsin, Tsviki Y. Hirsh, Tim T. Jäger, Manuel Morgano, Patrick Feng

**Affiliations:** 1 Forschungs-Neutronenquelle Heinz Maier-Leibnitz, 85748 Garching, Germany; 2 Los Alamos National Laboratory, Los Alamos, NM 87545 USA; 3 Idaho National Laboratory, Idaho Falls, ID 83415 USA; 4 Oak Ridge National Laboratory, Oak Ridge, TN 37830 USA; 5 University of California, Berkeley, CA 94720 USA; 6 Soreq Nuclear Research Center, 81800 Yavne, Israel; 7 Technische Universität Darmstadt, 64289 Darmstadt, Germany; 8 European Spallation Source ERIC, 22484 Lund, Sweden; 9 Paul Scherrer Institut, 5232 Villigen, Switzerland; 10 Faculty of Materials Engineering, Lund University, 22100 Lund, Sweden; 11 Sandia National Laboratories, Livermore, CA 94550 USA

**Keywords:** Characterization and analytical techniques, Imaging techniques

## Abstract

Energy-resolved fast-neutron radiography is a powerful non-destructive technique that can be used to remotely measure the quantity and distribution of elements and isotopes in a sample. This is done by comparing the energy-dependent neutron transmission of a sample with the known cross-sections of individual isotopes. The reconstruction of the composition is possible due to the unique features (e.g. resonances) in the cross-sections of individual isotopes. At short-pulsed (${\lesssim }$ 1 ns) neutron sources, such information is accessible via time-of-flight neutron imaging in principle, but requires a detector with nanosecond temporal resolution. Conventional neutron detectors can meet this requirement only by heavily compromising spatial resolution or efficiency. Here, we present a unique approach on fast neutron resonance radiography using a scintillator-based event-mode imaging detector at a short-pulsed neutron source, including first results on spatially mapped resonance profiles using MeV neutrons. The event mode approach applied in the presented detector allows recording of individual neutron interactions with nanosecond precision in time and sub-mm resolution in space. As a result, the entire available neutron energy spectrum can be measured for each pulse. At the same time, the use of a thick scintillator screen and lenses to focus the produced light results in a highly flexible field of view and a high interaction probability in the sensitive volume of the detector.

## Introduction

There are many situations in which it is desirable to measure the distribution of materials in a sample non-destructively. For example, if a sample is too valuable or dangerous to be damaged, or if a destructive technique would disturb the structure or process that should be measured. Energy-resolved neutron radiography is a method that can non-destructively and remotely (i.e. without the measuring equipment touching the sample) analyze distributions of elements and isotopes, which is one of the most fundamental properties of a material. This is done by measuring the neutron transmission spectrum through the sample. The distinctive variations in the energy-dependent neutron cross-section of different nuclides make it possible to deduce the composition from the transmission spectrum. Since the neutron cross-section is isotope-dependent, this method can also be used to extract the isotopic composition of a sample. For neutron radiography, these measurements are performed in a spatially resolved manner to get a map of the composition at different locations. Energy-resolved neutron radiography has already been demonstrated with epithermal neutrons (100 meV to 1 keV) for heavy elements and it has been used to create isotope maps^1–3^. As it focuses on the sharp resonance features that are common in the neutron cross-sections in this energy range, this is frequently called epithermal neutron resonance radiography. However, epithermal neutron resonance imagining is mostly sensitive to heavy elements, stable isotopes of light elements (up to Argon) typically do not exhibit resonances in this energy range. The only exception is $^{35}$Cl with a resonance at ${\sim }$ 400 eV.

Most distinctive features of the cross-sections of light elements are in the fast neutron energy range (from ${\sim }$ 100 keV to several tens of MeVs). To reliably differentiate between many elements and isotopes, the transmission has to be measured over a broad range of fast neutron energies. A well-established method of measuring such a broad energy range is the time-of-flight (ToF) technique. Applying the ToF method to fast neutrons requires a detector with high temporal resolution.

The basic techniques for energy-resolved fast-neutron radiography of light elements were developed in the mid 1980’s with a goal of explosives detection^4,5^. A 1D transmission profile was generated in these experiments by scanning the sample with a narrow neutron beam, and a 2D cut through the sample was calculated using tomographic reconstruction. Successful 2D radiography measurements have been carried out with a low spatial resolution of 1 cm, using an array of separated scintillators^6^. For higher resolution measurements, gated detectors^7,8^ have been successfully employed, but with the drawback that the detector can only use a small fraction of the incoming neutrons. Another approach has been to use a combination of a thin conversion layer and gaseous electron multipliers^9^. These detectors have a relatively low detection efficiency due to the limited thickness of the conversion layer. Overall, there is a lack of high spatial resolution detectors for fast-neutron ToF measurements that can use the incoming neutron beam efficiently. The detector system presented here has significant advantages over previous detectors, giving improved spatial resolution and time-resolution with relatively thick scintillators, and the ability to use a variety of scintillators depending on the needs of a particular measurement.

## Experimental setup

### 60R flight path at LANSCE

**Fig. 1:**
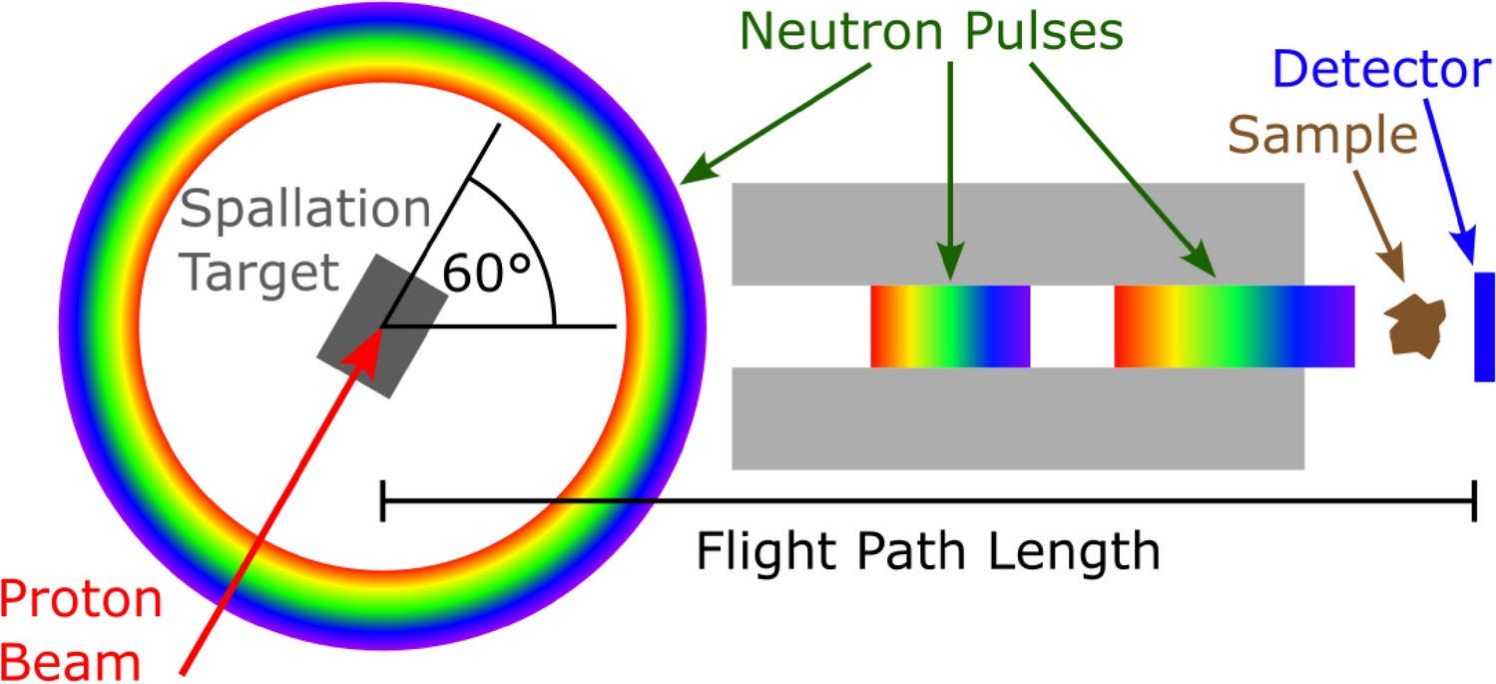
Schematic of the 60R beam line at LANSCE. Spallation neutrons are emitted by the target in all directions but only the ones emitted at an angle of 60$^\circ$ travel down the flight path. Different neutron energies are shown as colors from violet (higher) to red (lower).

**Fig. 2:**
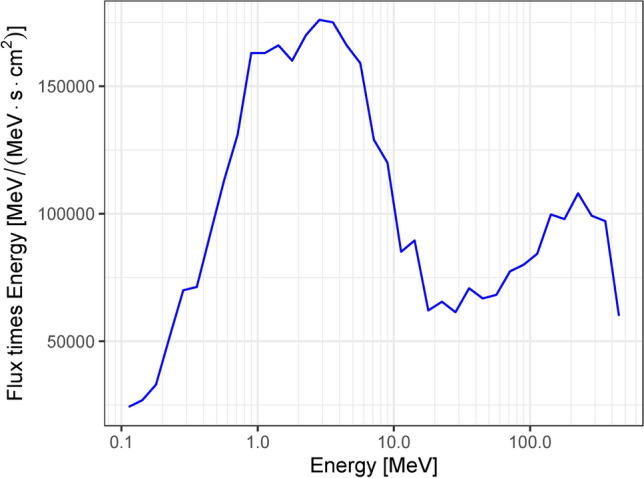
Neutron spectrum of the WNR 60R beamline^12^. The *y*-axis shows the flux times energy (also called flux per unit lethargy).

**Fig. 3:**
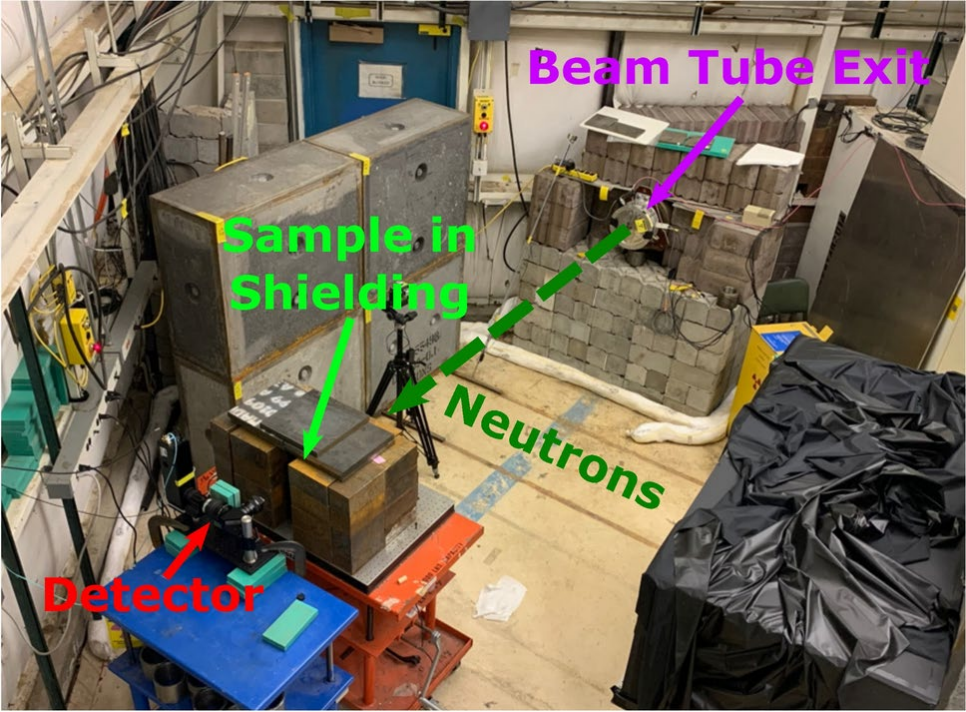
Photograph of the setup inside the experimental area at the 60R beamline. Collimated fast neutrons travel from the beam tube exit in the direction of the dashed dark green arrow through the sample and to the detector. The sample is shielded to suppress the contribution from scattered neutrons to the radiography as well as reduce the dose rate for the experimenters outside the experimental area.

Neutron imaging measurements were performed on the 60R beamline at the Weapon Neutron Research (WNR) facility located at the Los Alamos Neutron Science Center (LANSCE)^10,11^. Here, 800 MeV tightly-bunched (< 1ns) proton pulses, each containing on the order of $10^8$ protons, are delivered to a cylindrical tungsten spallation target of roughly 3 cm in diameter and 7.5 cm in length. When a proton pulse impinges upon the tungsten target, resultant spallation neutrons travel down the flight path unmoderated. The neutron energy spectrum depends on the neutron emission angle with respect to the incident proton beam direction^10^. For the 60R beamline, this angle is 60$^\circ$ (see Fig. 1) and the resulting spectrum is shown in Fig. 2. At a distance of ${\sim }$ 18 m from the source, the neutrons reach the experiment area where samples and detectors can be placed. For the measurements presented here, a position sensitive event-mode detector was used (see “Neutron detector” section). It was placed at a distance of ${\sim }$ 22 m from the spallation target. A schematic overview of the beamline is shown in Fig. 1. A photograph of the experiment area with the detector installed is shown in Fig. 3.

The absolute intensity of the beam at 60R can be measured via a calibrated fission ion chamber^13^. However, due to technical difficulties with the fission chamber during the measurements the absolute neutron flux is not provided. For relative comparison of neutron intensities between measurements, the number of pulses from the proton accelerator was recorded during each measurement. For the analysis of the data it is assumed that the average intensity of the pulses remains constant between different measurements.

### Neutron detector

**Fig. 4:**
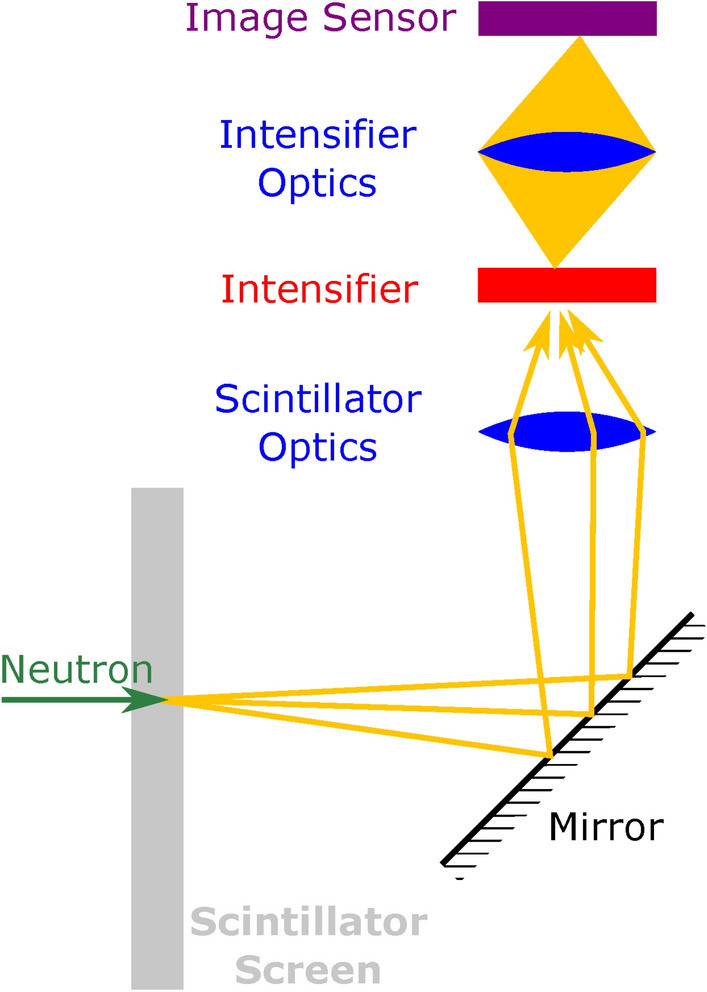
Illustration of the structure and working principle of the detector. Scintillation photons are shown as individual yellow arrows. The intensified light is shown as a solid yellow cone.

The detector used for the measurements was adapted from previous event-mode imaging detectors for thermal and epithermal neutrons^14,15^ by simply exchanging the scintillator. The structure of the detector is similar to what can be found in traditional scintillator-based neutron imaging detectors. The most important components are a scintillator screen, an image intensifier, an image sensor, a mirror, and two sets of lenses. A schematic overview of the components is shown in Fig. 4, and each is described in more detail below. All detector components are commercially available, and no special equipment is needed for assembling and operating the detector.

Incoming neutrons interact with the scintillator screen and produce scintillation photons. These photons are reflected by the mirror and focused on the image intensifier via the scintillator optics. The purpose of the mirror is to allow all subsequent components to be placed outside the direct neutron beam. This reduces noise from neutrons that are scattered from other camera components back to the scintillator screen and from direct interactions of gammas or neutrons with the intensifier or image sensor. The use of lenses as scintillator optics allows adapting the system to different fields of view with only minor modifications. The image intensifier is sensitive to single photons and produces a burst of light on its output whenever it is activated by a scintillation photon. The intensifier optics focus the light from the image intensifier output onto the image sensor. The burst of light produced by the image intensifier as a response to a single photon is bright enough to activate the pixels on the image sensor. In combination with the image intensifier, the image sensor is therefore sensitive to individual scintillation photons.

Three different scintillator screens were used. Two of them are so called Nanoguide scintillators^16^. The structure of these Nanoguide screens confines scintillation photons to move along a preferred axis inside the screen. For a conventional transparent scintillator, the thickness of the screen causes a spatial blurring effect in the recorded data as the scintillator optics can only sharply focus the light from a single plane within the scintillator onto the intensifier input. Scintillation light that is emitted at other locations inside the scintillator gets more blurred the further it is away from this focal plane. In a Nanoguide scintillator screen, scintillation photons created anywhere inside the scintillator travel along the Nanoguide direction to the surface of the scintillator. All light can therefore be considered to be emitted on the surface of the scintillator. This effectively eliminates the out-of-plane optical blur by focusing the scintillator optics on the surface of the Nanoguide screen. The remaining scintillator screen that was used in the measurement is an EJ-200 from Eljen Technology^17^. An overview of all scintillator screens is shown in Table 1. All the scintillators used here are activated via elastic scattering with the nuclei in the scintillator material. It is the recoil particle resulting from these interactions that creates the observed scintillation. Hydrogen is the most important contributor for this with respect to neutron detection due to the high hydrogen content in the scintillators and the high efficiency in energy transfer in the elastic neutron scattering process with hydrogen. The scintillators were mounted on the detector using 3D printed adapters, allowing easy exchange between scintillators of varying sizes and thicknesses.

**Table 1:** Overview of the scintillator screens used for the measurements.

Scintillator screen	Visible area	Thickness (mm)	FoV (mm)
EJ-200	7 mm × 7 mm	20	7
Nanoguide (small)	7 mm × 7 mm	12.7	7
Nanoguide (large)	100 mm × 100 mm	25.4	160

The FoV is indicated as the length of an edge of the square on the scintillator screen plane that is in view of the image sensor.

Two different scintillator optics were used to achieve different fields of view (FoVs). The first one uses two lens assemblies in an infinity-corrected configuration resulting in a FoV of 7 mm, and the second one uses a single lens assembly resulting in an FoV of 16 cm. Table 1 indicates which of the scintillator screens was used in combination with which FoV. It should be noted that only an area of 10 cm $\times 10$ cm of the large Nanoguide screen is visible and it therefore does not cover the full 16 cm FoV of the corresponding scintillator optics. Only the center region of data collected in this configuration therefore contains any meaningful information.

The image intensifier is a Photonis Cricket2 from Exosens^18^. The Cricket2 also includes the intensifier optics. The image sensor is a Timepix3 chip^19^ read out by a SPIDR evaluation board developed by Nikhef^20^. The Timepix3 chip has a grid of $256\times 256$ pixels with a size of 55 µm $\times$ 55 µm each. It measures pixel activation times with a resolution of 1.5625 ns. To support such a fine time resolution, the Timepix3 chip operates in event-mode. This means that it produces a list of pixel activations as its output (i.e. occurrences when enough light hits a pixel such that it becomes activated). Each entry in the list contains an x and y coordinate to identify the activated pixel and a timestamp. The duration for which the pixel was active (time over threshold) is provided as well, giving a measure of the amount of captured light for a given pixel activation. This event-mode approach is different from the frame-based mode used in most other imaging sensors, where a matrix of light intensities for all pixels is produced at a fixed rate, providing essentially a series of images.

### Event reconstruction algorithm

A neutron interaction in the scintillator screen typically results in multiple pixel activations on the Timepix3 chip. There are several reasons for this. First, the scintillation photons from a single neutron interaction are produced in the scintillator screen distributed over a region close to the interaction point, not only the interaction point itself. This is due to the finite range of reaction products (most importantly recoil protons). Dependent on the scintillator, the scintillation light can also be blurred due to not being emitted on the focal plane (see “Neutron detector” section). In addition, the scintillation photons are also distributed in time based on the decay of the scintillator. The image intensifier also produces a blur in the amplified light, resulting in even the signal of a single scintillation photon being distributed over multiple pixels.

**Fig. 5:**
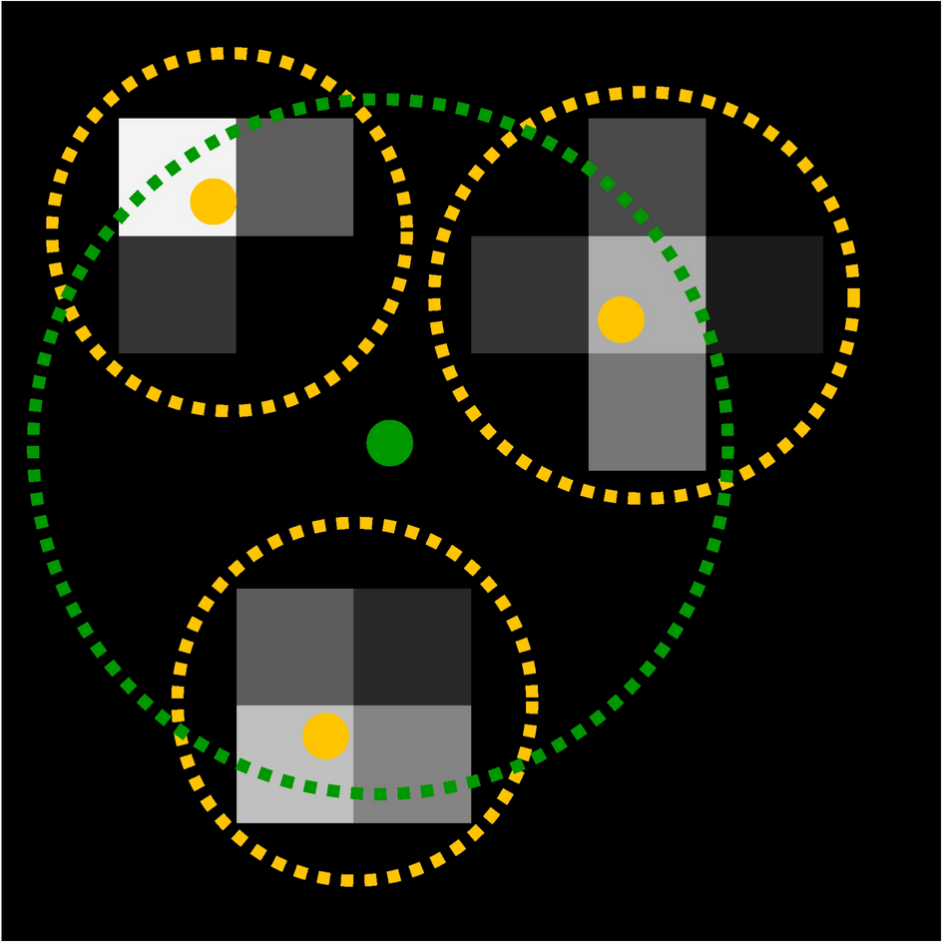
Simplified illustration of the event reconstruction steps (without the time domain). Pixel activations are shown as a grey scale matrix indicating the time over threshold. Yellow dashed lines show pixel activation groups with the reconstructed scintillation photon as a yellow dot. The green dashed line illustrates all scintillation photons belonging to the same group with the green dot as the reconstructed neutron interaction location.

The goal of the event reconstruction algorithm is to separate the signals from different events and reconstruct the original interaction positions of the neutrons with the scintillator screen in time and space. It is based on the event reconstruction described by Losko et al.^14^. The reconstruction happens by essentially following the physical processes inside the detector backwards. In the first step, the scintillation photons impinging upon the image intensifier are reconstructed from the pixel activations. In the second step, the neutron interactions are reconstructed from the scintillation photons. Both steps are described in detail below. Figure 5 illustrates the entire process, disregarding the time component for a better overview. In reality, the additional temporal component leads to the photons of a single event arriving sequentially, inherently simplifying the separation of individual scintillation photons from a single event.

For the scintillation photon reconstruction, all pixel activations are separated into groups (yellow dashed lines in Fig. 5). Two pixel activations are put in the same group if their spatial distance is less than or equal to a parameter *s*_px_ and their temporal separation is less than or equal to a parameter $t_\text {px}$. Pixel activations that do not have to be placed in the same group according to this rule are placed in separate groups. All scintillation photon reconstructions in this paper are performed with *s*_px_ px and $t_\text {px} = 50$ ns. Only groups containing 2 or more pixel activations are considered to belong to scintillation photons. All groups consisting only of a single pixel activation are considered to be noise and are disregarded for further processing. The scintillation photon position (yellow dot in Fig. 5) is approximated as the average of the position of all pixel activations in the group, weighted by their time over threshold. This averaging provides sub-pixel resolution for the reconstructed scintillation photons. The timestamp of the earliest pixel activation in the group is taken as the arrival time for the reconstructed scintillation photon.

For the neutron interaction reconstruction, all the reconstructed scintillation photons are separated into groups (green dashed line in Fig. 5) using the same algorithm as for the pixel activations: Two photons are grouped together if their spatial distance is less than or equal to *s*_phot_ and their temporal separation is less than or equal to *t*_phot_. Only groups containing a number of scintillation photons above or equal to a threshold *k* are considered to be the signal from a neutron interaction. Other groups are considered to be noise (e.g. from gamma rays or intensifier dark counts) and are disregarded. The values of the parameters for the neutron interaction reconstruction are changed based on the detector configuration. They can be found in Table 2. The neutron interaction position is approximated as the average of the positions of all photons in the group, each photon having the same weight (green dot in Fig. 5). The arrival time of the earliest scintillation photon is taken as the interaction time of the neutron.

**Table 2:** Maximum spatial distance *s*_phot_, maximum temporal distance *t*_phot_, and threshold *k* for the reconstruction of neutrons from scintillation photons for the different detector configurations.

Scintillator	FoV [mm]	*s*_phot_ [px]	*t*_phot_ [ns]	*k* [1]
EJ-200	7	40	15	3
Nanoguide	7	30	30	3
Nanoguide	160	3	15	2

The value of *s*_phot_ is shown in physical pixels of the Timepix3 chip.

It is worth pointing out that conventional scintillator-based neutron imaging detectors sum up all the light produced by the scintillator. Any image acquired with these detectors is therefore blurred by the spread of the scintillation light detected for each neutron event (e.g. due to the range of the recoil proton or distance to the focal plane). By using the event-mode reconstruction algorithm described above, the result is blurred by the uncertainty in the reconstructed position instead. As long as the events are identified correctly, this is always at least as precise as the equivalent scintillation light spread blur in conventional scintillator-based neuron imaging detectors, and especially in high resolution setups can be significantly more precise. Aside from integrating the scintillator light emission induced by neutrons, conventional neutron imaging detectors also include any light from noise sources e.g. light leaks. In contrast, using the correct parameters for the event reconstruction algorithm, noise can be filtered out, resulting in cleaner data. This also means that (depending on the exact setup and targeted precision), dark image (detector noise) subtraction, which is typically performed for conventional scintillator-based imaging systems, is usually not required as the background in the raw data is already small enough to be negligible.

### Energy sensitivity using time of flight

ToF is a technique where the neutron energy is measured via the time it takes the neutron to travel from the source to the detector. If the distance from the source to the detector is known, this time can be used to determine the velocity of the neutron and therefore also its kinetic energy. The following equation describes the relation between time and energy (taking into account relativistic effects for fast neutrons):

1 $$\begin{aligned} E_k = \left( \frac{1}{\sqrt{1 - \frac{L^2}{t^2c^2}}} - 1\right) m_n c^2 \end{aligned}$$

Here, *t* is the ToF, *L* is the distance between source and detector, *c* is the speed of light in vacuum, $E_k$ is the kinetic energy of the neutron, and $m_n$ is the neutron mass. One thing to note is that the reconstruction of the neutron energy from the ToF becomes more uncertain at higher energies. This can be seen from the derivative with respect to time:

2 $$\begin{aligned} \frac{dE_k}{dt} = \frac{(2E_kc^2m_n + E_k^2)^{3/2}}{c^3m_n^2L} \end{aligned}$$

The derivative increases more than linearly with the neutron energy. For a fixed uncertainty in the ToF, e.g. due to inaccuracies in the arrival time measured by the detector, the absolute and relative error in the calculated energy increases for higher neutron energies. This can be mitigated by increasing the distance *L* between source and detector. Fast-neutron ToF applications therefore usually require good timing accuracy as well a long distance between source and detector to achieve the desired energy resolution.

Time of flight is typically measured by using a source that emits bursts of neutrons in short pulses and a time-sensitive detector. While traveling down the flight path, neutrons will begin to separate in time based on their energies. Higher energy neutrons from a single pulse will arrive at the detector at an earlier time than lower energy neutrons from the same pulse. Figure 1 illustrates this based on the 60R beamline at the WNR. As long as the time between pulses is long enough to avoid overlap (the fastest neutrons of one pulse do not arrive at the detector before the slowest neutrons of the previous pulse), the arrival time at the detector uniquely defines the pulse the neutron belongs to. With that, the emission time at the source $t_0$ is known as well. Based on the detection time of the neutron $t_D$, the time of fight $t = t_D - t_0$ can be calculated. The duration of each pulse and the timing accuracy of the detector are usually the most important factors when considering the accuracy of the measured ToF. The high timing accuracy needed for fast-neutron ToF measurements therefore mandates short neutron pulses from the source.

Mechanical choppers are frequently used in thermal neutron ToF measurements to either create neutron pulses from a continuous source or shorten pulses from an inherently pulsed source to achieve better timing resolution. However, the much higher timing accuracy typically required for an adequate energy resolution in the fast neutron range makes it infeasible to use choppers to create or modify the pulse structure. Inherently short-pulsed sources such as the WNR at LANSCE are therefore essential for fast-neutron ToF measurements.

## Results and discussion

### Flight path calibration

**Fig. 6:**
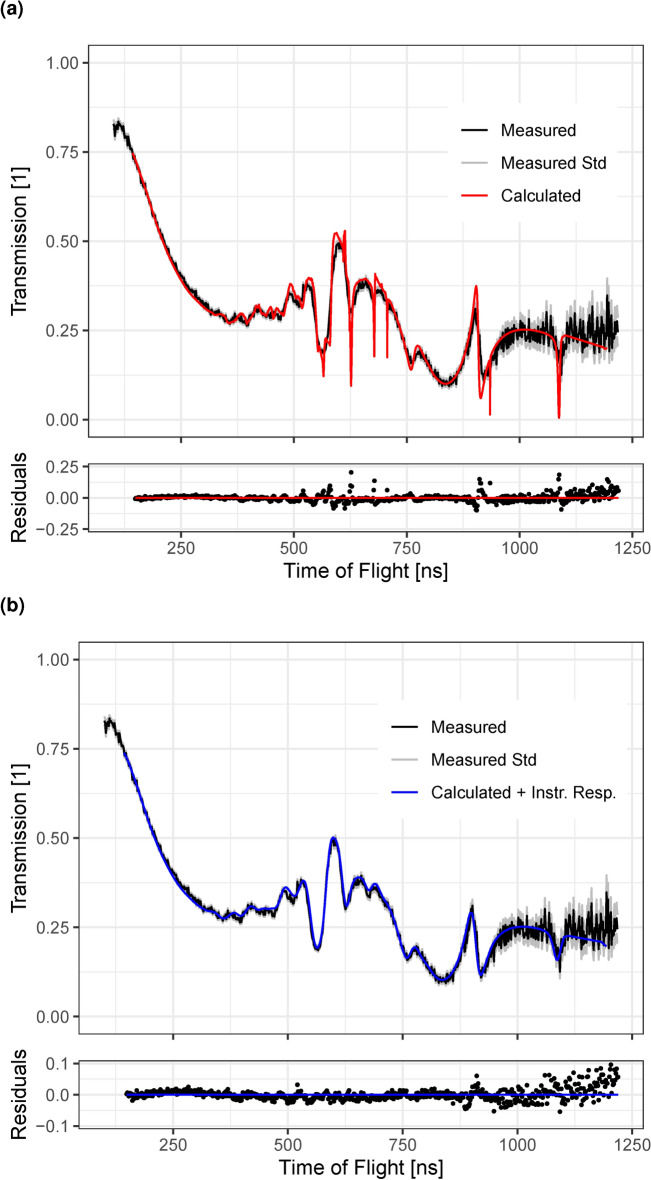
(**a**) Comparison of the measured transmission as a function of ToF through the graphite block to the transmission calculated from literature using the fitted flight path length and $t_0$ offset. The displayed standard deviation only takes into account counting statistics. (**b**) The same data but with the instrument response function applied.

The emission time of the neutrons at the target $t_0$ was provided as an electronic signal by the facility to the detector. However, there is a small, setup-dependent timing offset $\Delta t_0$ on the signal, causing the detector to measure $t_0 + \Delta t_0$. To calculate the neutron energy accurately, this offset has to be known and corrected for. Additionally, the flight path length *L* has to be known. The two parameters were determined by measuring the neutron transmission through a graphite block and fitting the transmission profile calculated from the known carbon cross-section data to it. This was done using the same data as in “Carbon with different scintillators” section and the total cross-section (N,TOT) for natural carbon (C-0) from the ENDF/B-VII.1 library^21^. The transmission calculation is performed by using the Beer-Lambert law with the total macroscopic neutron cross as the attenuation coefficient i.e. without considering the possibility of scattered neutrons being detected. The literature carbon cross-section has a high energy resolution and therefore includes much sharper features than the measured transmission. To account for this in the fitting process, the timing precision of the setup is modeled by a Gaussian distribution as the instrument response function. A convolution of the calculated transmission profile with this instrument response function is used for the comparison with the measured data. The standard deviation $\sigma _t$ of the Gaussian distribution is included as an additional parameter in the fit. The EJ-200 scintillator screen measurements were used for this calibration. The best fit was obtained for a flight path length $L = (21.64\pm 0.010)$ m, $\Delta t_0 = (-48.8\pm 0.3)$ ns, and $\sigma _t = (6.84\pm 0.17)$ ns (see Fig. 6), resulting in a reduced $\chi ^2$ of 2.2.

The calculated transmission in Fig. 6b agrees well with the measured data, indicating that a Gaussian distribution can be used to describe the uncertainty in the timing of the setup well and that the timing precision is ${\sim }$ 6.8 ns. From Eq. (2), this results in a corresponding accuracy in the neutron energy of ${\sim }$ 2% at 5 MeV, which is the center of the region with prominent features. This Gaussian distribution will be applied to theoretical transmissions throughout the remainder of this work to account for the temporal instrument response.

### Carbon with different scintillators

The carbon transmission was measured with each of the two 7 mm FoV scintillators (see Table 1). Two measurements were conducted for each scintillator: one without any sample in the beam (open beam), and one with a 10.2 cm thick graphite block covering the entire FoV of the scintillator screen. The exposure time for each measurement was 10 min. This is a short time when compared to measurements of other imaging detectors at the same beamline, e.g. Noam et al. recorded for 2 h for a graphite ToF spectrum with a single pixel being about equivalent in size to the full 7 mm $\times 7$ mm FoV used here^6^.

**Fig. 7:**
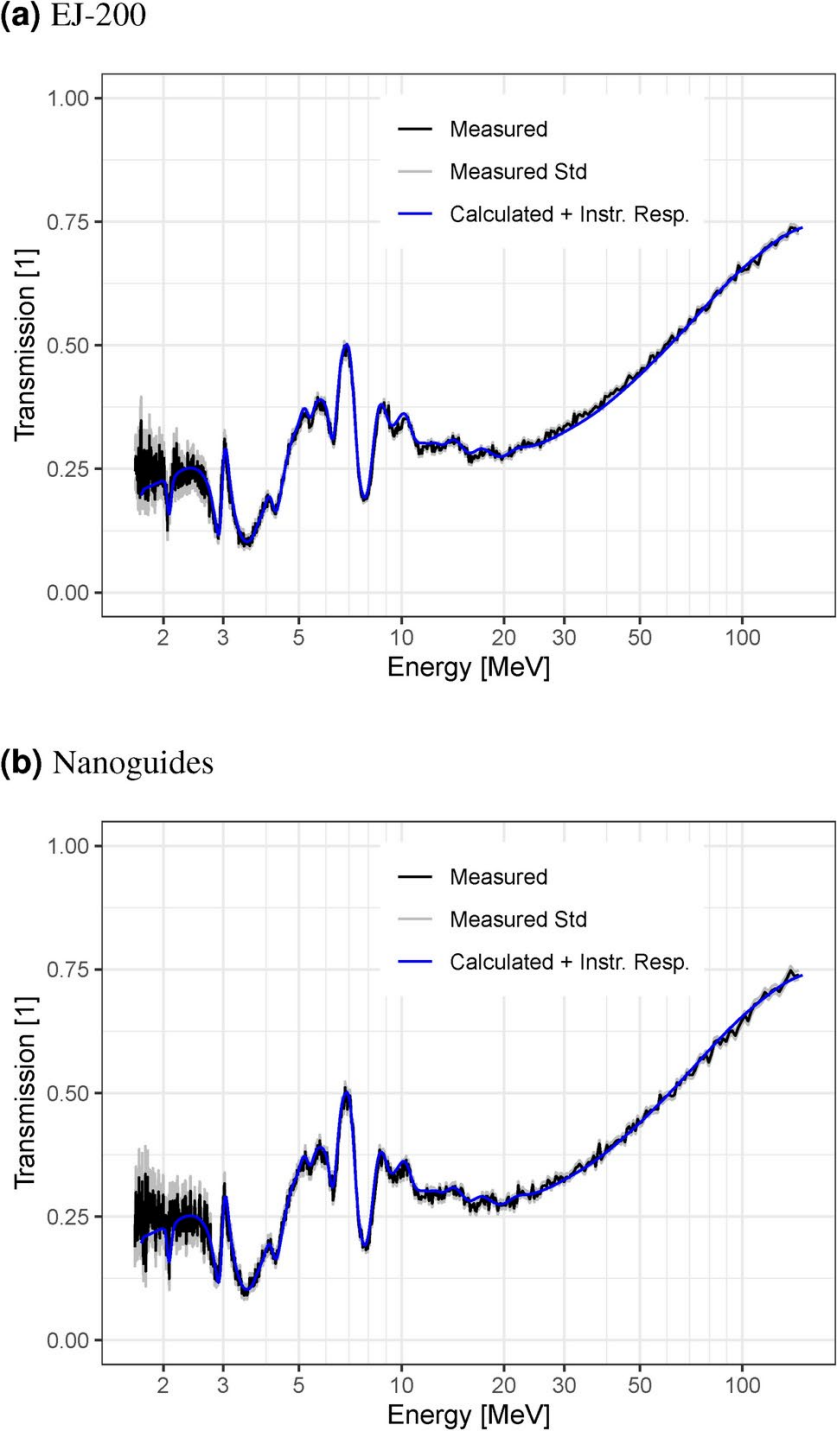
Energy-dependent transmission through a graphite block measured with different scintillator screens. The transmission calculated from ENDF data and with the temporal instrument response function applied is shown as a comparison. The grey lines indicate a single standard error, only taking into account uncertainties from counting statistics.

The detected events are binned into 1.5625 ns time bins, and summed up over the entire detection area. The number of events in each bin is normalized by dividing it with the number of accelerator pulses during the measurement. The normalized number of events in each time bin for the sample measurement is divided by the number of events in the same bin of the open beam measurement to obtain the transmission. No background subtraction is performed for either measurement. The equivalent neutron energy for each time bin is calculated according to Eq. (1). The results for both scintillators are shown in Fig. 7. The measured values are compared to the transmission calculated for the graphite block using the total cross-section (N,TOT) for natural carbon (C-0) from the ENDF/B-VII.1 library^21^, applying the temporal instrument response function (identical for both scintillators) derived in “Flight path calibration” section. The transmission calculation is performed by using the Beer-Lambert law with the total macroscopic neutron cross as the attenuation coefficient i.e. without considering the possibility of scattered neutrons being detected.

The data collected with the different scintillators is quite similar. In both cases, the measured value matches the transmission calculated from ENDF data closely. This shows that the detector is well suited for fast-neutron ToF imaging with both scintillators. The well resolved and precise transmission indicates excellent capabilities for quantitative measurements of the composition of samples.

### Test image

To assess the capabilities of the detector for imaging applications, a transmission image of two samples, a piece of glass (SiO$_2$ optical lens) and a graphite block, was measured using the large Nanoguide scintillator screen with a 16 cm FoV (see Table 1). The different composition of the two objects (SiO$_2$ for the lens and C for the graphite) should lead to different shapes of the energy-dependent transmission, providing a first test for the method to distinguish between materials. With the larger FoV, the gamma flash became a problem in this measurement as it saturated the image intensifier for a significant time. To prevent this, the image intensifier was turned off for a short period of time at the beginning of each pulse using its built-in gating input. One measurement was conducted with the samples in the neutron beam and one without the samples (open beam). Each of the two measurements ran for ${\sim }$ 30 min.

**Fig. 8:**
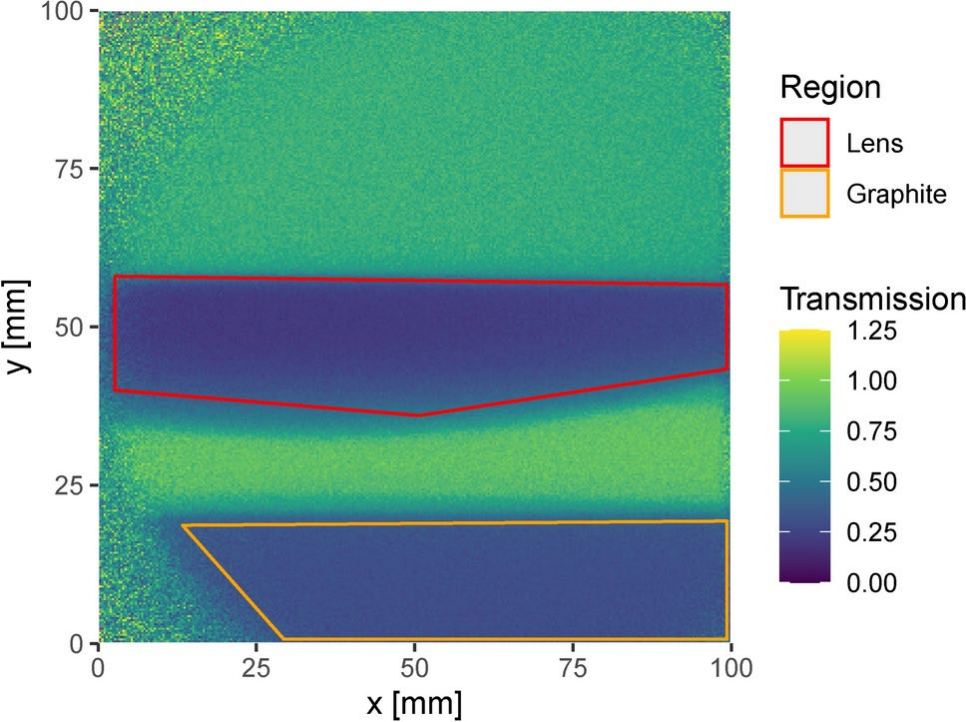
Transmission image integrated over the entire neutron spectrum of the lens and graphite block measured with the large FoV setup. The image is cropped to the large Nanoguide scintillator region as data outside of this does not carry any (meaningful) information. The beam is slightly off-center and the upper and lower left corners are therefore not illuminated The two regions used to analyse the sample-specific transmission are marked.

The events of each measurement are binned in 1.5625 ns time bins and $512\times 512$ spatial pixel bins with a size of $\sim$ 0.2 mm $\times$ 0.2 mm. The number of events in each bin is normalized by dividing it with the number of accelerator pulses during the corresponding measurement. The normalized number of counts in every bin for the measurement with the samples is then divided by the normalized number of events in the same bin for the open beam measurement to get the transmission. No background subtraction was performed for either measurement. Since the scintillator screen was smaller than the FoV, the edges do not contain any meaningful information. Figure 8 shows the transmission in the scintillator region averaged over the entire ToF spectrum.

**Fig. 9:**
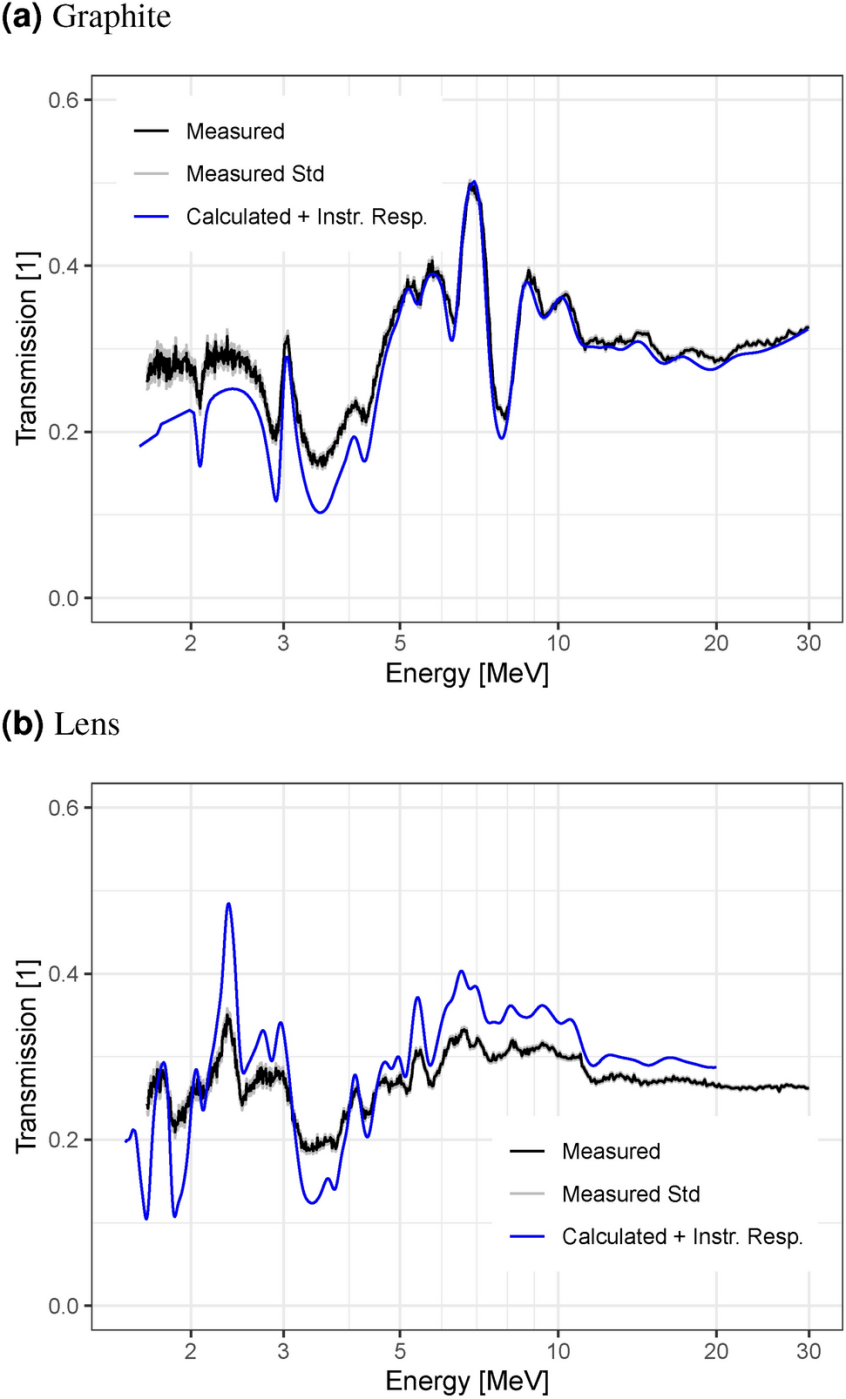
Transmission profiles measured with the large FoV setup, averaged over the graphite block region (**a**) and the lens region (**b**) as indicated in Fig. 8. Profiles calculated from ENDF data with the instrument response function applied are shown in blue as a comparison.

The ToF dependent transmission is averaged for a region with the lens in the beam and a region with the graphite block in the beam respectively. The two regions are marked in Fig. 8 and the averaged transmission profiles are shown in Fig. 9. The transmission calculated from ENDF library data for the respective materials is shown for comparison. The graphite block is the same one as was used for the measurements described in “Carbon with different scintillators” section and the same ENDF library data is used to calculate its theoretical transmission. The lens mostly consists of SiO$_2$ and the total cross-sections (N,TOT) for natural silicon (Si-0) from the ENDF/B-VI library^22^ and for oxygen-16 (O-16) from the ENDF/B-VIII.0 library^23^ are used to calculate the theoretical transmission. Although the thickness of the lens varies over the image, the calculated geometry is simplified to a homogeneous thickness of 10 cm at a density of 2.4 g/cm^2^. It therefore only allows a qualitative comparison of the prominent features. Absolute transmission values as well as its variation differ due to the different geometries in measurement and calculation. The calculation of the theoretical transmission is performed by using the Beer-Lambert law with the total macroscopic neutron cross as the attenuation coefficient i.e. without considering the possibility of scattered neutrons being detected.

The samples were positioned closer to the detector when compared to the measurements presented in “Carbon with different scintillators” section, leading to a significant amount of scattering from the samples into the scintillator screen. This can be observed from the transmission greater than 1 measured in the detector region between the samples (see Fig. 8).

Figure 9 shows the measured transmission in the graphite region following the calculated data quite well. The match is not as good as in the Nanoguide data presented in “Carbon with different scintillators” section, but all the features except some very narrow resonances are visible. The absolute values show significantly more deviation, especially in the region between 1 MeV and 5 MeV. The reason for the additional discrepancies when compared to the results in “Carbon with different scintillators” section are likely the increased sample scattering contribution.

To include the sample scattering in the calculated transmission, the scattering background contribution to the transmission $B_T$ was modeled as an exponential function of the neutron energy $E_k$:

3 $$\begin{aligned} B_T(E_k) = b \cdot e^{-a \cdot E_K} \end{aligned}$$

**Fig. 10:**
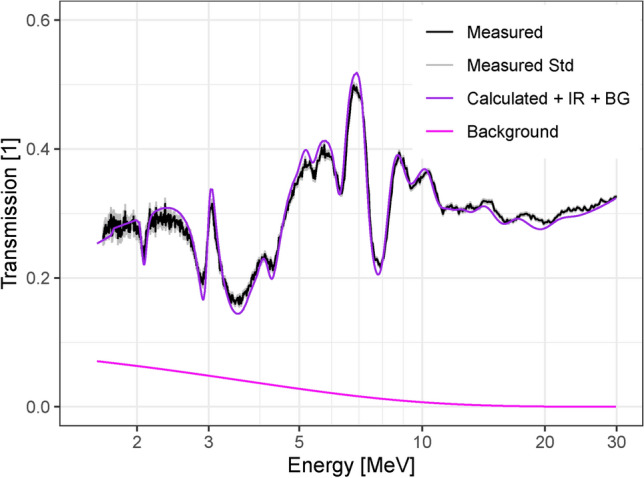
Comparison of the calculated transmission (purple) when including the fitted background contribution (magenta) with the measured transmission (black). The calculated transmission is shown with the instrument response function (IR) applied.

This background was added to the calculated transmission for the graphite region and fitted to the measured transmission with *a* and *b* as parameters. The best fit was obtained for $a = (0.273\pm 0.014)$ MeV^−1^ and $b = 0.109\pm 0.006$, resulting in a reduced $\chi ^2$ of 11. Figure 10 shows the result of the fit. The transmission shows a good agreement with the discrepancy in the absolute values at lower energies being almost completely eliminated by the introduction of the fitted background.

The SiO$_2$ and C materials are clearly distinguished by the measured transmission profile. This indicates that the setup can be used not only to differentiate between materials but also to quantitatively measure the atom composition of a sample based on the unique energy dependent variations in the fast neutron cross sections of the different nuclides.

## Conclusion and outlook

The measurement results show that the presented setup is well suited for energy-resolved fast-neutron imaging. The timing resolution of 6.8 ns is enough to resolve the main resonance features in the total neutron cross sections of the carbon and SiO $_2$ samples. The absolute values of the measured transmission with the graphite sample match the calculated data well. The deviation observed with the graphite sample close to the detector are likely due to scattering and can be corrected by fitting subtracting a simple background function. Here, the theoretical transmission was known in advance but similar techniques can also be used for unknown samples by conducting a coarse resolution measurement at a large distance to the detector to establish a clean baseline for the transmission of the sample.

By using a scintillator screen, event reconstruction, and flexible binning, the detector can achieve a high number of pixels over the entire FoV. As it relies on lenses to focus the scintillation light, different FoVs are possible with only small changes to the setup. This unique combination of temporal and spatial resolution in a flexible design makes the presented design a promising candidate for various fast-neutron imaging applications, especially fast-neutron ToF imaging. The detector can make use of the entire neutron pulse and the thick scintillator options provide a high interaction probability for fast neutrons. This efficient use of neutrons is mirrored in the relatively short acquisition times. A full energy resolved image including the required open beam was recorded in ${\sim }$ 1 h. With the high resolution and the short acquisition times, the setup is also ideal for 3-dimensional composition reconstruction via tomography.

While this paper focused on the resonance imaging modality of this detector, further measurements are underway to investigate its characteristics, such as efficiency, spatial resolution, and other detector parameters. These will be discussed in future publications. Another route for future investigations is to image compound materials and test the sensitivity of the detector signal with respect to composition changes. In addition, the accuracy of different methods for reconstructing the composition from the measured signal can be tested.

As the detector concept is relatively new, there are many areas where significant improvements can be expected in the near future. Some directions currently being investigated are the scintillator, the intensifier optics, and the event reconstruction algorithm.

## Data Availability

Data can be made available by the corresponding author upon reasonable request.
